# Revolutionizing Autoimmune Kidney Disease Treatment with Chimeric Antigen Receptor-T Cell Therapy

**DOI:** 10.34133/research.0712

**Published:** 2025-05-22

**Authors:** Rui Gu, Jiayi Shen, Jiayu Zhang, Jianhua Mao, Qing Ye

**Affiliations:** ^1^ Department of Nephrology, Children’s Hospital, Zhejiang University School of Medicine, Hangzhou 310052, China.; ^2^ Department of Laboratory Medicine, Children’s Hospital, Zhejiang University School of Medicine, Hangzhou 310052, China.

## Abstract

Autoimmune kidney diseases (AIKDs) depict a range of disorders involving immune-mediated damage to the kidneys, where conventional biologic therapies involving monoclonal antibodies often prove insufficient because of persistent autoreactive B cell reservoirs in lymphoid organs and inflammatory tissues. The appearance of chimeric antigen receptor (CAR)-T cell therapies targeting B cells has shown transformative potential, with recent clinical trials showing the remarkable efficacy of anti-CD19 CAR-T cells in achieving profound B cell depletion, reducing immune complex deposition, and ameliorating renal inflammation in AIKDs. While these results highlight the potential of CAR-T cell therapy in facilitating immune reset and overcoming treatment resistance, further clinical investigations are imperative to establish its long-term safety and sustained therapeutic benefits. This review synthesizes current evidence on CAR-T cell applications in AIKDs, discusses critical considerations for clinical translation, identifies existing limitations and challenges, and proposes strategic directions for therapeutic optimization and advancement.

## Introduction

Autoimmune kidney diseases (AIKDs) are driven by disruption of immune tolerance and aberrant autoimmune responses, leading to renal inflammation and tissue damage. They encompass systemic autoimmune disease-related nephritis [e.g., lupus nephritis (LN) and anti-neutrophil cytoplasmic autoantibody-associated glomerulonephritis (AAGN)], organ-specific autoantibody-mediated conditions (e.g., anti-glomerular basement membrane disease), and some primary kidney diseases closely related to the autoimmune mechanism [e.g., membranous nephropathy (MN), idiopathic nephrotic syndrome (INS), and immunoglobulin A nephropathy (IgAN)] [1,2]. B cell-driven humoral immune dysregulation plays a central role in their pathogenesis, as evidenced by the clinical success of B cell-targeted monoclonal antibody therapies [3,4]. However, a substantial proportion of patients exhibit persistent or relapsing disease activity, likely due to incomplete depletion of pathogenic B cell subsets.

Targeted antigen-specific chimeric antigen receptor (CAR)-T cells represent an innovative category of genetically modified T cells, with the unique ability to identify and target specific antigens precisely. Their remarkable success in treating hematologic cancers has ushered in a novel era of immunotherapy. Notably, the regulatory approval of CAR-T cell therapies that target CD19, a marker stated on the surface of B cells, has catalyzed advancements in applying CAR technology beyond oncology, fostering pioneering translational research in areas such as autoimmune disorders, with systemic lupus erythematosus (SLE) being a prime example [4–6], myasthenia gravis [7], rheumatoid arthritis [8], and multiple sclerosis [9], and has demonstrated the durable efficacy of high specificity for the removal of B cells and the preservation of remission. This finding offers a new therapeutic option for other systemic autoimmune diseases, including those of the kidney. Although the inflamed kidney may act as a tertiary lymphoid structure with high in situ infiltration of B cells [10–12], currently available preclinical models and case reports continue to demonstrate the powerful ability of CAR-T cell therapy to achieve deep B cell clearance and immune reset.

In this review, we outline the critical function of B cells in the pathogenesis of AIKDs and focus on the therapeutic potential of CAR-T cell therapy. We summarize the available evidence, including findings from preclinical studies and ongoing and planned CAR-T cell clinical trials. Furthermore, we discuss key considerations of CAR-T cell therapy prior to large-scale clinical rollout, summarize potential limitations and challenges, and suggest future directions for optimization and improvement.

## Targeting B Cell Therapy for AIKDs

**Fig. 1.**
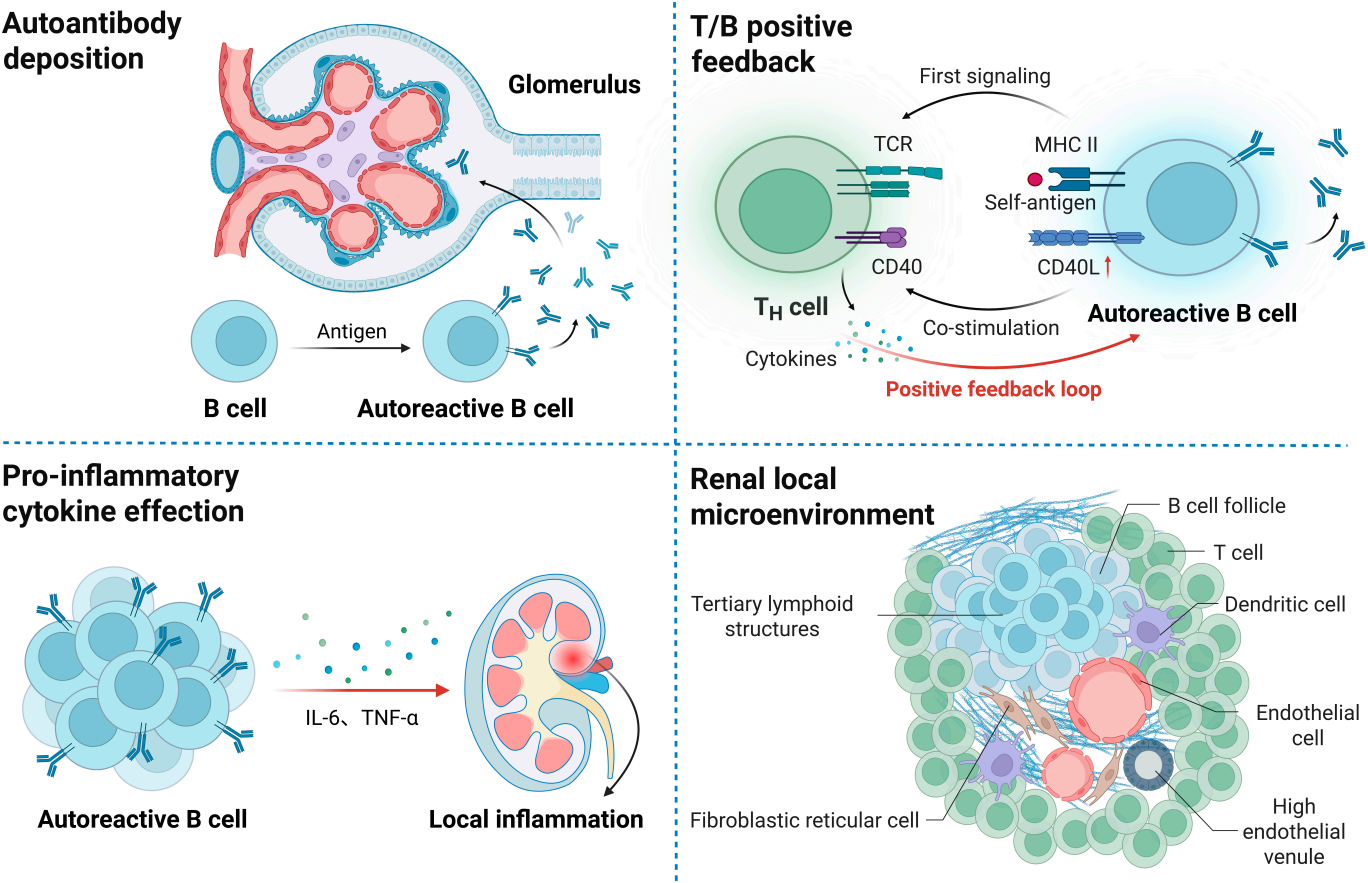
Pathogenic mechanisms of B cells in autoimmune kidney diseases (AIKDs). T_H_ cell, T helper cell; TCR, T cell receptor; MHC, major histocompatibility complex; IL-6, interleukin-6; TNF-α, tumor necrosis factor-α.

The pathogenic mechanisms of B cells in AIKDs involve multiple levels of aberrant activation and dysregulated immune regulation (Fig. 1). The most critical aspect is the escape of autoreactive B cells from immune tolerance checkpoints, resulting in their abnormal activation as well as subsequent secretion of pathogenic autoantibodies against specific self-antigens, such as DNA, nuclear proteins, glomerular basement membrane proteins, and podocyte surface antigens [13–16]. Second, B cells present self-antigens via major histocompatibility complex (MHC) class II molecules and provide costimulatory signals to activate autoreactive CD4^+^ T cells. The activated T cells subsequently secrete pro-inflammatory cytokines, further enhancing B cell function and up-regulating costimulatory pathways, establishing a bidirectional positive feedback loop that is progressively amplified under conditions of immune tolerance breakdown and chronic inflammation, ultimately driving disease progression [17]. Moreover, autoreactive B cells produce cytokines like interleukin-6 (IL-6), further amplifying local inflammation [3,18]. Finally, chronic inflammation may reshape the renal immune microenvironment, with high endothelial vein aberrant expression of chemokines recruiting circulating lymphocytes (T/B cells, macrophages) to infiltrate the kidneys, inducing the formation of ectopic tertiary lymphoid structures, and exacerbating immune attacks on renal target tissues. Abnormal interactions between infiltrating immune cells and tissue-resident cells (fibroblasts, endothelial cells) lead to a vicious cycle of chronic inflammation-promoting fibrosis, which ultimately results in irreversible glomerular and interstitial injury [19,20]. B cell depletion therapies act by targeting aberrant B cell functions, thereby helping to halt or slow the progression of AIKDs.

**Fig. 2.**
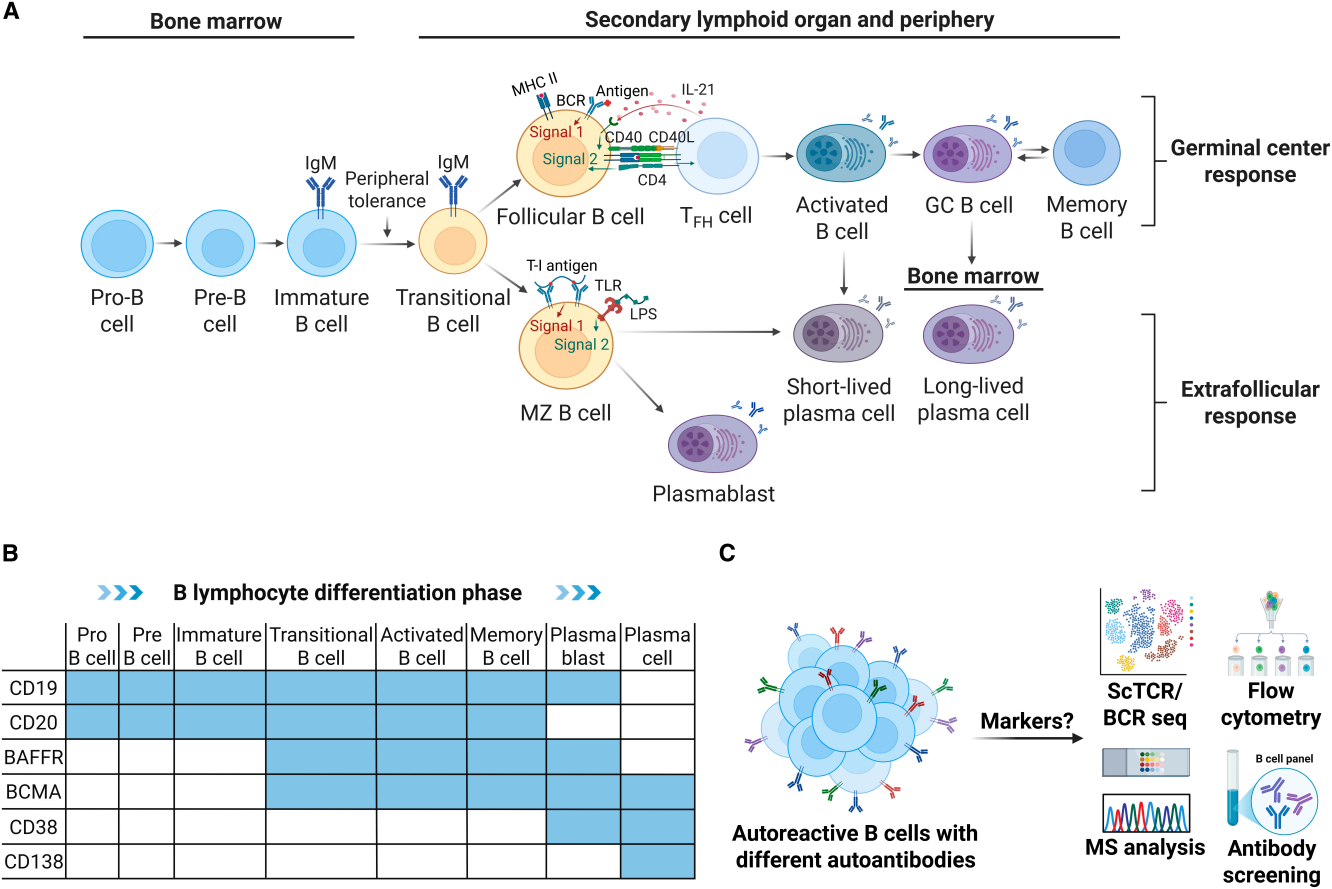
(A) B cell differentiation and development process. (B) B cell surface antigen markers at different phases. (C) Technical methods for the precise characterization of surface markers of pathogenic B cell clones. MZ, marginal zone; GC, germinal center; BAFFR, B cell activating factor receptor; BCMA, B cell maturation antigen; BCR, B cell receptor; MS, mass spectrometry; Ab, antibody; T_FH_, T follicular helper.

A deeper understanding of B cell development, differentiation, immune responses (Fig. 2A), and membrane-specific markers (Fig. 2B) is essential for designing precise therapeutic strategies targeting specific B cell subsets. B cell responses can manifest as either germinal center or extrafollicular responses. The extrafollicular response generates plasmablasts and short-lived plasma cells (PCs) that secrete low-affinity antibodies, a phenomenon frequently observed in infections and autoimmune diseases [21,22]. Increased circulating short-lived PCs have been reported in patients with SLE [23], and in INS, autoreactive extrafollicular B cell clones may serve as a primary source of podocyte autoantibodies [24]. In contrast, the germinal center response primarily involves long-lived PCs (LLPCs) and memory B cells [25]. In autoimmune diseases, inflammatory tissues, including the kidney, can sustain LLPCs, leading to the continuous generation of high-affinity pathogenic autoantibodies and the localized inflammation [26–30].

B cell-targeted biologics have been approved for the treatment of LN, MN, AAGN, and refractory INS. Rituximab (RTX) induces rapid depletion of circulating CD20^+^ B cells, whereas belimumab achieves a relatively mild reduction in B cells by blocking survival signal transduction mediated by B cell activating factor. However, in certain patients, the therapeutic efficacy of these agents remains suboptimal, with frequent relapses observed. A major limitation is the widespread distribution of B cell reservoirs within mucosal immune tissues, while monoclonal antibodies are largely confined to the bloodstream and exhibit limited tissue penetration. This poses a challenge for the complete eradication of tissue-resident B cells, including B cell maturation antigen (BCMA)-positive LLPCs. Studies indicate that residual B cells remain in the lymphoid tissues and renal interstitium of LN patients after RTX treatment [31]. Notably, a study on myasthenia gravis demonstrated that during B cell reconstitution after RTX therapy, newly emerging B cell clones still included autoreactive populations [32]. In addition, memory B cells and PCs are not dependent on B cell activating factor for survival, which may lead to resistance to belimumab in SLE patients [33]. These findings suggest that RTX fails to completely eliminate pathogenic autoreactive B cells, which is akin to merely trimming visible weeds while retaining their roots. In contrast, CAR-T cell therapy can uproot these pathogenic B cell clones, achieving true immune reset. As living drugs, CAR-T cells can proliferate and migrate to various lymphoid tissues and organs, sustaining B cell depletion and inducing long-time remission. In patients receiving anti-CD19 CAR-T cell therapy for autoimmune diseases, B cells are completely depleted in secondary lymphoid organs [34]. A systematic comparison of CAR-T cell therapy and conventional biologics is summarized in Table 1.

**Table 1.** Comparison between CAR-T cell therapy and conventional biologics in AIKDs

Indicators	CAR-T cell	Conventional biologics
Mechanism	Genetically engineered modified T cells directly recognize and kill specific target cells (e.g., CD19^+^ B cells and BCMA^+^ plasma cells)	Recognition of specific molecular antigens on the surface of B cells (e.g., CD20 and BAFF) and targeted clearance through cytotoxicity and other effects
Therapeutic features	High response rate, deep remission	Moderate-high response rate, predominantly partial remission
Durability	Long-term remission, potential curability	Requires repeat infusion therapy, high relapse rate, potential for drug resistance
Side effects	CRS, ICANS, hematologic toxicity, increased risk of infection, nephrotoxicity	Infusion-related reactions, hematologic toxicity, increased risk of infection
Treatment cost	High cost of single infusion treatment, reduced treatment costs when long-term remission is achieved	Higher cumulative cost of repeated infusions
Individualized treatment	Target-specific customization and personalized optimization for patient’s B cell phenotype, pathological clonal expansion, antigenic mutations	Often the target is fixed and the “one drug fits all” model is difficult to optimize for the individual

CAR, chimeric antigen receptor; BCMA, B cell maturation antigen; BAFF, B cell activating factor; CRS, cytokine release syndrome; ICANS, immune effector cell-associated neurotoxicity syndrome

Compared with CD20, CD19 covers a broader spectrum of the B cell lineage and remains the most extensively utilized CAR-T cell therapy target to date [35,36]. In contrast, BCMA exhibits a highly restricted expression pattern within the antibody-secreting lineage, specifically in plasmablasts and PCs. CAR-T cell therapies aimed at BCMA have shown significant effectiveness in treating relapsed or refractory multiple myeloma, and recent translational efforts have extended BCMA-targeted strategies to autoimmune diseases characterized by pathogenic PC activity [37,38]. Dual-targeting approaches combining BCMA and CD19 leverage the synergistic effects of PC depletion and precursor B cell targeting, potentially enhancing therapeutic durability and preventing antigen escape.

However, most current CAR-T cell therapies rely on fixed antigen targets for global B cell depletion, an indiscriminate approach that may result in the loss of nonpathogenic B cell populations, resulting in humoral immunodeficiency and prolonged immunosuppression. Thus, next-generation CAR-T cell therapies must focus on “just pulling weeds”—precisely identifying and selectively removing pathogenic B cell clones while maximizing immune homeostasis (Fig. 2C). To achieve this goal, advanced technologies like single-cell immune repertoire sequencing and flow cytometry can be employed to delineate the phenotypic characteristics and specific molecular markers of autoreactive B cell clones. This information can inform the design of personalized CAR constructs, ultimately enabling precision immune intervention for autoimmune diseases.

## Fundamentals and Overview of CAR-T Cell Therapy

**Fig. 3.**
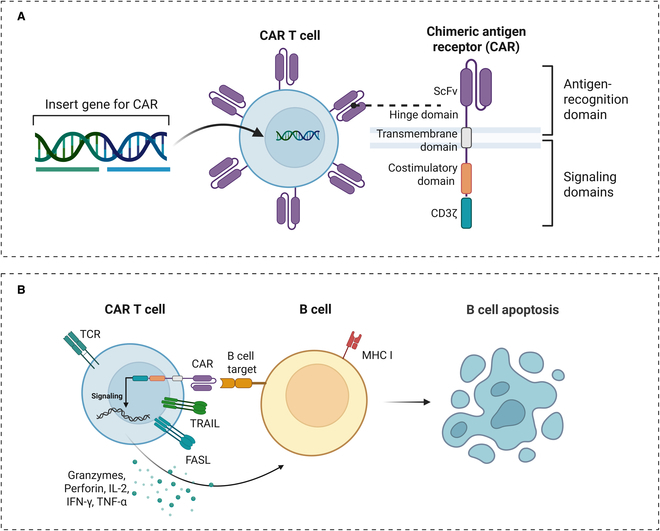
(A) Typical structural design of chimeric antigen receptor (CAR) constructs. (B) Mechanism of action of CAR-T cells targeted to kill B cells. TRAIL, TNF-related apoptosis-inducing ligand; FASL, Fas ligand; IFN-γ, interferon-γ.

CAR is a genetically engineered fusion protein, and the typical CAR structure contains 5 key functional domains (Fig. 3A): an extracellular single-chain fragment variable that targets antigens, a hinge region that enhances receptor flexibility, a transmembrane structural domain, a costimulatory molecule (e.g., CD28 or 4-1BB), and a T cell activation signaling domain (CD3ζ) [39,40]. CAR-T cells kill target cells independently of MHC recognition and trigger the death receptor pathway mainly by targeting B cell surface antigens (Fig. 3B): The release of tumor necrosis factor (TNF)-related apoptosis inducing ligand with Fas ligand induces caspase-3-dependent apoptosis in target cells, whereas the perforin granule enzyme system directly disrupts the target cell membrane structure by forming transmembrane pores [41]. In addition, proinflammatory cytokines (e.g., IL-2, interferon-γ, and TNF-α) secreted by CAR-T cells can further activate macrophage-mediated antibody-dependent cellular phagocytosis, resulting in a cascade effect that synergistically removes pathologic B cells [42].

**Fig. 4.**
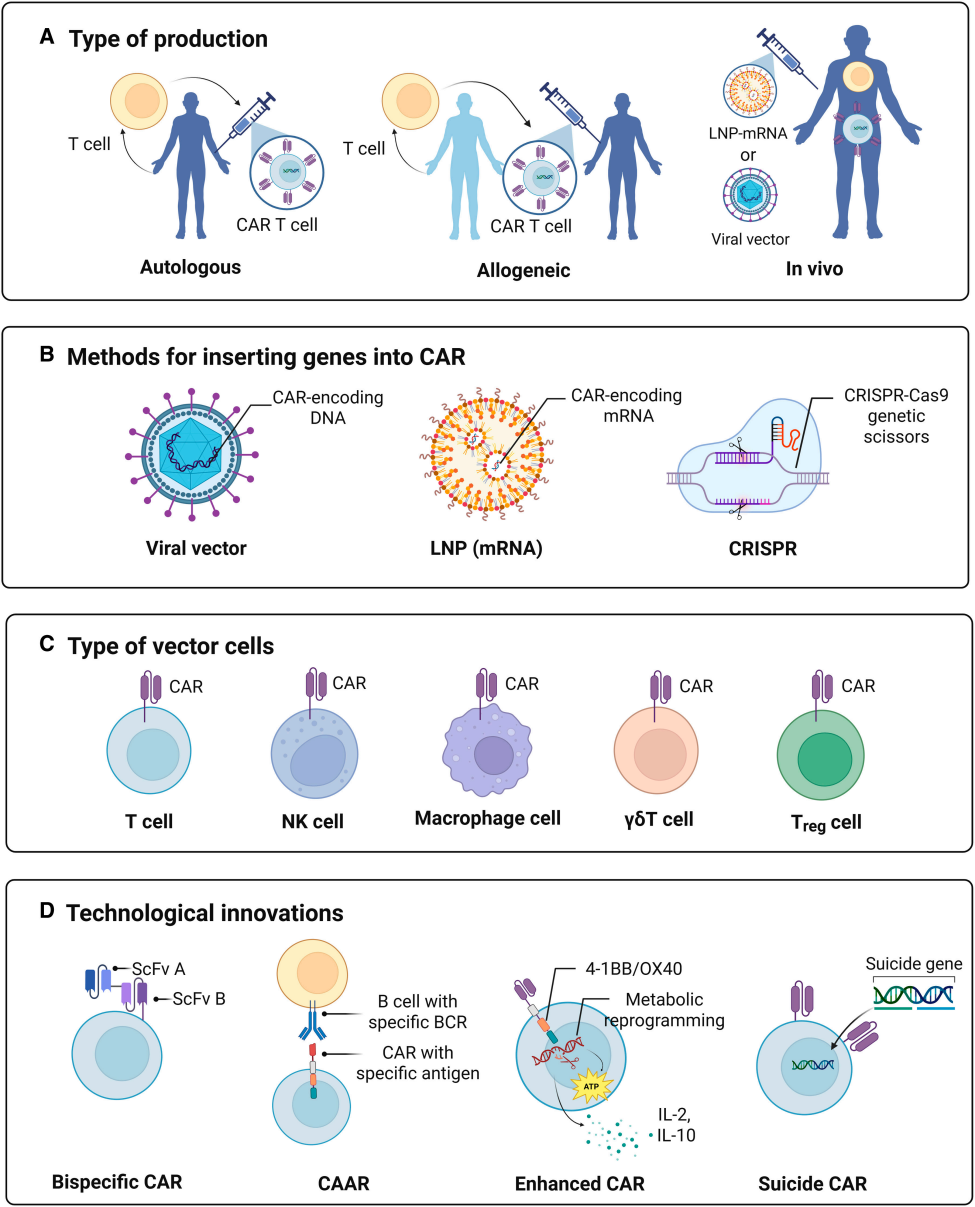
(A) Several different methods for producing CAR-T cells. (B) Different methods for transducing genes into CARs. (C) Different vector cell types for CAR. (D) Several innovative designs for CAR-T cells. Bispecific CARs can simultaneously target 2 antigens to enhance therapeutic breadth; CAARs eliminate pathogenic B cells by expressing disease-specific autoantigens; enhanced CARs integrate costimulatory signaling and metabolic reprogramming to improve cellular persistence and function; suicide CARs incorporate controllable genes to enable precise regulation and reduce toxicity risk. LNP, lipid nanoparticle; mRNA, messenger RNA; NK, natural killer; T_reg_, regulatory T; ScFv, single-chain variable fragment; CAAR, chimeric autoantibody receptor.

Current production strategies for CAR-T cell therapies have undergone a significant shift (Fig. 4A), expanding from traditional autologous infusion to allogeneic universal CAR (UCAR)-T cells and introducing an in situ engineering platform that uses lipid nanoparticle (LNP) delivery of messenger RNA (mRNA) encoding CARs to directly reprogram T cells in vivo [43–45]. The innovative methods have significantly decreased patient waiting times, simplified manufacturing processes, and lowered the costs of treatments. To transform T cells from donors into UCAR-T cells, CRISPR gene editing technology is frequently used to disable endogenous T cell receptors and MHC-like genes. This step is crucial for preventing allogeneic rejection [46,47]. The initial clinical trial utilizing UCAR-T cell therapy in 3 patients with systemic sclerosis and necrotizing myositis demonstrated promising efficacy and safety [48]. Nonetheless, there remains a need for further data regarding long-term efficacy and safety. Particularly in patients with autoimmune diseases, the prevalent immune dysregulation still has the risk of leading to immune rejection. This may significantly increase additional healthcare expenditures, including prolonged hospitalization, intensified use of immunosuppressants, and management of associated complications, thus partially offsetting the original cost advantage of UCAR-T products. The development of CAR-T therapies is in a transitional stage of seeking an optimal solution between “cost efficiency” and “safety and control”, and the balance between efficacy and affordability will be the key to their clinical translation. In terms of gene delivery systems, retroviral vectors are still predominant, and although long-lasting and stable expression of CARs can be achieved, the auto-immunogenicity and randomized integration of viral vectors increase the risk of insertional mutagenesis. To overcome this shortcoming, multiple advanced nonviral approaches such as LNP delivery vectors not only avoid the risk of genome integration but also enable transient expression with controlled regulation [49,50]. CRISPR-based targeted integration technology precisely inserts CAR genes into predefined “safe harbor” sites, improving safety while enhancing the functional durability and phenotypic stability of CAR-T cells [51,52] (Fig. 4B).

Moreover, the development of innovative vectors for CAR-T cell therapy aims to address the limitations of traditional T cells, thereby enhancing the applicability and safety of this therapeutic approach (Fig. 4C). In parallel, CAR- natural killer cells have emerged as promising alternatives. These cells significantly lower the risk of graft-versus-host disease and cytokine release syndrome (CRS) because of their MHC-unrestricted recognition mechanism and inherent immune characteristics [53–55]. CAR-macrophages possess naturally efficient tissue permeability and phagocytic antigen presentation dual functional functions, which may have potential advantages in targeting local inflammation modulation and removing aberrant immune complexes in the kidney [56]. γδ T cells [57] and regulatory T (T_reg_) cells [58], on the other hand, utilize their pan-specific recognition pattern and immunomodulatory properties to expand their targeting scope while enhancing safety. In addition to dual-target CAR structural designs, which are already quite widely used in autoimmune diseases, other advanced and innovative CAR technologies are being continuously developed (Fig. 4D). Chimeric autoantibody receptor (CAAR)-T cells, also referred to as “trans-CAR T” cells, are designed to eliminate autoreactive B cells with high precision by expressing disease-specific autoantigens as their extracellular domains. This antigen-directed approach enables the selective targeting of pathogenic B cell clones while sparing nonpathogenic B cell populations, thereby minimizing systemic immunosuppression [59–61]. Recent studies have demonstrated the therapeutic potential of CAAR-T cells in experimental models of MN [62] and LN [63], highlighting the critical importance of precise antigen identification for optimizing treatment efficacy. Enhanced CAR-T cell therapies demonstrate improved persistence and survival in inflammatory microenvironments, largely due to optimized costimulatory signals such as 4-1BB or OX40 replacing traditional CD28. CRISPR-based reprogramming of T cell metabolism and cytokine secretion has also been shown to enhance efficacy while reducing the required dose [64–68]. The suicide CAR, on the other hand, realizes spatiotemporally specific regulation. By implanting suicide-inducible genes (e.g., Caspase9), suicide CARs can activate suicide signaling with specific small-molecule drugs when severe adverse effects are detected, leading to rapid programmed apoptosis of CAR-T cells [69,70]. These innovations provide multidimensional solutions to overcome the bottlenecks of CAR-T cell therapies in terms of insufficient durability, off-target toxicity, and immune microenvironment resistance. However, these emerging technologies for CAR in AIKDs are still in the early exploratory stage, and more studies are needed to validate their safety and efficacy before large-scale clinical accessibility.

## Clinical Progress and Prospects of CAR-T Cell Therapy in AIKD Patients

CAR-T cell therapy has shown significant effectiveness in treating hematologic malignancies and autoimmune diseases. This success has driven further research into CAR-based therapies for AIKDs, with ongoing clinical trials aiming to evaluate the potential of CAR-T cells in modulating immune responses, alleviating renal injury, and improving patient outcomes (Table 2).

**Table 2.** Ongoing clinical trials of CAR-T cell therapy for AIKDs

Disease	CAR target(s)	Cell source	Manufacturing type	CAR-T product name	Clinical trial number	Phase and estimated enrollment	Study duration
Lupus nephritis (LN)	CD19	T cell	Autologous	N/A	NCT06585514	I/II, 18	2024–2025
		T cell	Autologous	KYV-101-001	NCT05938725	I/II, 32	2023–2026
		T cell	Autologous	KYV-101-003	NCT06342960	I/II, 32	2022–2029
		T cell	Autologous	Rapcabtagene autoleucel	NCT06581198	II, 144	2024–2030
		T cell	Autologous	CNCT19	NCT05930314	I, 12	2023–2025
		T cell	Autologous	YTB323	NCT05798117	I/II, 24	2023–2026
		T cell	Autologous	CABA-201	NCT06121297	I/II, 12	2024–2027
		T cell	Autologous	SYNCAR-001 + STK-009 (Orthogonal IL-2)	NCT06544330	I, 42	2024–2041
		T cell	Allogeneic	SC291	NCT06294236	I, 36	2024–2028
		T cell	Autologous	KYV-101	NCT06152172 (not yet recruiting)	I, 24	2024–2028
		NK cell	Allogeneic	NKX019-102	NCT06557265	I, 21	2024–2027
		T cell	Allogeneic	ATA3219	NCT06429800 (not yet recruiting)	I, 26	2024–2029
	CD20	T cell	Allogeneic	ADI-001	NCT06375993 (not yet recruiting)	I, 40	2024–2027
	BCMA	T cell	Autologous	PRG-1801	NCT06277427	N/A, 24	2024–2027
		T cell	Autologous	PRG-1801	NCT06497387	I, 30	2024–2028
	CD19/CD20	T cell	Autologous	IMPT-514	NCT06153095	I/II, 30	2024–2027
		T cell	Autologous	Zamto-Cel	NCT06708845 (not yet recruiting)	I, 48	2025–2026
	CD19/BCMA	T cell	Autologous	BH002	NCT06350110	I/II, 75	2024–2025
		T cell	Autologous	PRG-2311	NCT06497361	I, 30	2024–2028
		T cell	Allogeneic	N/A	NCT06681337 (not yet recruiting)	I, 10	2024–2025
		T cell	Autologous	FKC288	NCT06285279	I, 24	2024–2028
	CD19/CD20/CD22	T cell	Autologous	LCAR-AIO	NCT06653556 (not yet recruiting)	I, 34	2024–2029
ANCA-associated glomerulonephritis	CD19	T cell	Autologous	N/A	NCT06508346	Observational, 12	2024–2027
		T cell	Autologous	N/A	NCT06056921	I, 24	2023–2026
		T cell	Autologous	RD06-04	NCT06548607	I, 20	2024–2027
		T cell	Autologous	RD06-04	NCT06549296	I, 12	2024–2027
		T cell	Allogeneic	SC291	NCT06294236	I, 36	2024–2028
		T cell	Autologous	RD06-04	NCT06548620 (not yet recruiting)	I, 18	2024–2027
		T cell	Allogeneic	BRL-301	NCT05859997	N/A, 15	2023–2025
		T cell	Autologous	N/A	NCT06420154 (not yet recruiting)	I, 9	2024–2027
		T cell	Autologous	KYV-101	NCT06590545 (not yet recruiting)	I/II, 8	2025–2027
		T cell	Autologous	KYV-101	NCT06152172 (not yet recruiting)	I, 24	2024–2028
		T cell	Autologous	ATMP	NCT06685042 (not yet recruiting)	I/II, 8	2024–2025
	BCMA	T cell	Autologous	PRG-1801	NCT06277427	N/A, 24	2024–2027
	CD19/CD20	T cell	Autologous	IMPT-514	NCT06462144	I, 36	2024–2026
	CD19/BCMA	T cell	Autologous	BH002	NCT06350110	I/II, 75	2024–2025
		T cell	Autologous	FKC288	NCT06285279	I, 24	2024–2028
	CD19/CD3E	T cell	Autologous	N/A	NCT06373081	N/A, 6	2024–2026
Multidrug-resistant nephrotic syndrome (MDR-SRNS)	BCMA/CD70	T cell	Autologous	N/A	NCT06553898	I, 18	2024–2027
IgA nephropathy (IgAN)	CD19	T cell	Autologous	IM19	NCT06690359 (not yet recruiting)	I, 12	2024–2026
Membranous nephropathy (MN)	CD19	T cell	Autologous	IM19	NCT06690359 (not yet recruiting)	I, 12	2024–2026
	CD19/BCMA	T cell	Autologous	FKC288	NCT06285279	I, 24	2024–2028
Immune nephritis	CD19/BCMA	T cell	Autologous	N/A	NCT05085418	I, 9	2021–2024
	CD19	NK cell	Autologous	KN5501	NCT06469190	I, 36	2024–2026

ANCA, anti-neutrophil cytoplasmic antibody; N/A, not applicable; NK, natural killer

### Anti-CD19 CAR-T cells

In preclinical research, anti-CD19 CAR-T cell therapy has demonstrated effectiveness in removing autoreactive B cells and reducing renal IgG immunodeposition and inflammatory cell infiltration in a mouse model of SLE [71,72]. Additionally, this therapy has been effective in preventing and delaying the onset of SLE in mice [73]. These promising results have paved the way for further clinical investigations. A young female patient with severe refractory SLE and grade IIIA active LN received anti-CD19 CAR-T cell therapy. This intervention led to the depletion of circulating B cells, a rapid decrease in disease activity, the resolution of proteinuria, and a significant improvement in renal damage. There were no relapses or treatment-related adverse events during 18 months of follow-up [4]. The same research group subsequently treated and analyzed 5 patients with refractory adult SLE in depth, all of whom showed significant improvement in symptoms, disappearance of renal inflammatory manifestations, and immunophenotypic analyses that revealed that the reconstituted B cells were predominantly initial B cells [5]. However, one patient continued to have low-level proteinuria at 3 months, suggesting that CAR-T cell therapy may have limited efficacy in cases where irreversible renal damage has already occurred. More recent follow-up data have shown that 8 patients with refractory SLE combined with LN maintained disease inactivity, pathogenic autoantibody deficiency, and stable levels of vaccine-associated protective antibodies for 29 months [6].

Compared to adults, pediatric SLE is often characterized by more aggressive disease phenotypes, a higher incidence of renal involvement, and an increased burden of treatment-related morbidity [74]. Building upon the promising outcomes observed in adult cohorts, CD19-CAR T cell therapy has emerged as a potentially valuable strategy for children with severe, treatment-refractory disease. Preliminary clinical reports have demonstrated favorable tolerability and notable therapeutic responses in pediatric patients. For example, in a 15-year-old girl with severe SLE and class IV LN, CAR-T cell therapy minimized renal inflammation and preserved remaining renal function [75]. We also reported 2 pediatric patients with refractory SLE who received CAR-T therapy. Both exhibited complete B cell depletion, significant reductions in autoantibody titers, and clinical improvement within 4 to 5 months post-infusion. One of these patients with grade IV LN underwent a repeat renal biopsy, which showed a reduction in acute inflammatory indices, although chronic damage remained, highlighting the challenges of reversing established structural injury [76]. While these findings underscore the feasibility and potential efficacy of anti-CD19 CAR-T cells in pediatric SLE, further research is essential to optimize dosing strategies, evaluate long-term safety, and address developmental and immunologic considerations unique to the pediatric population. These efforts will be critical to positioning CAR-T therapy as a transformative option in the management of childhood-onset SLE.

Encouraged by the initial success of CD19-targeting CAR-T cell therapy in SLE and LN, there is growing interest in extending this approach to other antibody-mediated AIKDs, particularly AAGN. Preclinical data have provided early proof of concept for this approach. In a murine model of anti-neutrophil cytoplasmic autoantibody-associated vasculitis, anti-CD19 CAR-T cells were shown to effectively migrate to the kidneys and continuously eliminate B cells, and prevent the development of AAGN. However, clinical evidence remains absent. To bridge this gap, we have initiated a prospective, 2-year follow-up study (NCT06508346) to evaluate the safety and efficacy of anti-CD19 CAR-T cell therapy in patients with refractory AAGN, marking a critical step toward clinical translation. Clinical trials are now exploring CD19 CAR-T cell therapy in other AIKDs, such as IgAN and MN, where the results are highly anticipated.

### Anti-BCMA CAR-T cells

Notably, complete exhaustion of autoantibodies was not observed with anti-CD19 CAR-T cell therapy, either in clinical trials of LN [5,6] or in preclinical models of AAGN [77]. This suggests that CD19-negative but BCMA-expressing pathogenic PCs—especially LLPCs—may persist and continue to drive disease. LLPCs have been implicated in refractory LN and are associated with disease severity [78]. There are reports that anti-BCMA CAR-T therapy can serve as a salvage treatment in patients with other autoimmune diseases who failed anti-CD19 CAR-T infusion, suggesting that PC depletion may improve outcomes in refractory cases [79]. However, their depletion also poses risks, as LLPCs are critical for maintaining protective immunity. Prior studies have shown that extensive depletion of LLPCs can rapidly reduce vaccine-induced IgG levels [80]. Based on this, multiple clinical trials have begun to explore the feasibility of using BCMA as a standalone target or in combination with CD19 for CAR-T cell therapy in AIKDs. A phase I clinical study of BCMA-CD19 CAR-T cells in SLE patients showed a marked reduction in anti-Sjögren syndrome B antibody autoantibodies following treatment, suggesting successful elimination of pathogenic antibodies predominantly produced by LLPCs [37]. In some cases, although hepatitis B surface antibody titers dropped to nearly undetectable levels, they rapidly recovered after revaccination, indicating that immune memory may be preserved in certain patients. Compared to CD19 CAR-T therapy alone, BCMA-CD19 CAR-T treatment resulted in a more pronounced decline in IgG levels, but only mild, manageable infection events were observed. Nevertheless, thoroughly evaluating the long-term safety of LLPC-targeting strategies remains essential, particularly in balancing the risk–benefit trade-off between pathogenic antibody clearance and preservation of protective immunity. Engineering CARs to selectively recognize pathogenic rather than protective LLPCs may represent a key strategy to address this challenge.

### CAR-T_reg_ cells

T_reg_ cells maintain immune tolerance by preventing the activation and proliferation of autoreactive T cells [81]. Although polyclonal T_reg_ therapies have shown efficacy in treating autoimmune diseases, their nonspecific immunosuppression raises safety concerns [82]. Recent studies have introduced CAR technology into T_reg_ cells to produce antigen-specific CAR-T_reg_ cells, which can accurately recognize and immunomodulate disease-associated antigens, thereby restoring immune homeostasis and attenuating tissue inflammatory damage [58,83]. Reduced T_reg_ cell numbers and defective T_reg_ function are closely associated with the development and progression of various AIKDs [84–86]. Preclinical studies have found that secondary transfer of T_reg_ cells reduces B cell counts and attenuates symptoms in a mouse model of chronic graft-versus-host disease with SLE [87]. Prevention of AIKD progression by T_reg_ cells has been demonstrated across various animal models, including LN [88], antiglomerular basement membrane disease [89], and IgAN [90]. Therefore, CAR-T_reg_ cell therapy for AIKDs is highly feasible, and the future development of a targeted delivery system for CAR-T_reg_ cells is expected to achieve precise regulation of the local immune microenvironment in the kidney. However, there are some potential concerns before CAR-T_reg_ cells can be used in the clinic, as contaminated effector T cells may be transduced by T_reg_ cells, which may exacerbate autoimmune perturbations. Prolonged suppression of immune surveillance functions may lead to increased susceptibility to infection or malignancy. In addition, CAR-T_reg_ may undergo functional exhaustion or transdifferentiation into an effector T cell phenotype in the inflammatory microenvironment.

## Barriers and Advanced Strategies for CAR-T Cell Therapy in AIKDs

While CAR-T cell therapy holds significant promise for patients with multidrug-resistant and refractory AIKDs, several key considerations and challenges still need to be addressed in its clinical application. A thorough evaluation of these factors is essential to achieve the best possible therapeutic outcomes for patients. Continued research in these areas will help improve the overall feasibility and widespread adoption of this innovative treatment strategy.

### Patients, optimal selection of therapeutic targets, and timing of treatment

CAR-T cell therapy in AIKDs is still in the exploratory stage, and the key to its success lies in 3 dimensions. The first dimension involves the precise identification of potential beneficiaries, necessitating a departure from traditional clinicopathological classifications. This involves retrospective analysis of patient subgroups that respond well to CAR-T cell therapy and the establishment of a precise immunophenotyping and cell therapy efficacy prediction model on the basis of the single-cell multiomic features of the disease, both before and after treatment. The second dimension focuses on the precise selection of dynamic targets for CAR-T cells. Patients with various AIKDs may display distinct immune features and antigen expression patterns at different stages of the disease. For example, the early stage of MN is dominated by podocyte antigens such as PLA2R/THSD7A, but there may be antigen escape and increased expression of other novel antigens in relapsed patients [91]. In addition, disease typing based on different interferon pathways can be used to guide target selection in LN patients, and type I/II LN patients may have different B cell subpopulations driving them [92,93]. The third dimension is the timing of the intervention window. At present, CAR-T cell therapy is utilized primarily for patients who are resistant to multiple drugs. These patients often have intricate medical histories with prolonged treatment courses. The combined side effects of various treatments, along with disease relapse, can lead to irreversible kidney damage, such as glomerulosclerosis and fibrosis, especially when the immune system’s ability to adapt is diminished. However, owing to the potential nephrotoxicity associated with CAR-T cell therapy, most clinical trials require a glomerular filtration rate of at least 30 ml/min/1.73 m^2^. This requirement may cause some patients to miss the optimal timing for treatment and limit the benefits they can gain from CAR-T cell therapy. Intervening as early as possible during the acute, immunologically active phase of the disease, before extensive fibrosis occurs, may be advantageous for maximizing treatment efficacy. Additionally, clinical trials in patients with aggressive B cell lymphoma have shown that CAR-T cell therapy may offer superior clinical benefit as a first-line treatment compared with conventional approaches [94,95]. Whether CAR-T therapy can be used in the early stage of AIKDs remains an important question. Exploring its potential to improve efficacy and prognosis in patients at low to intermediate risk will be a major focus of future research. The establishment of a standardized risk–benefit scoring system related to CAR-T cell therapy may be beneficial in the future to guide clinical decision making. In addition, comprehensive pretreatment assessment of organ function, including repeated renal biopsy, is essential to ensure that patients with residual renal function can tolerate CAR-T cell therapy.

### Management of adverse reactions associated with CAR-T cell therapy

Early common adverse reactions associated with CAR-T cell therapy include CRS and immune effector cell-associated neurotoxicity syndrome (ICANS). CRS is characterized by a severe systemic inflammatory response resulting from the in vivo overactivation and proliferation of CAR-T cells. These cells produce large amounts of cytokines, such as IL-6 and TNF-α, which can disrupt the integrity of the blood–brain barrier and subsequently trigger a neurotoxic response [96,97]. However, clinical data suggest that the incidence of CRS and ICANS in patients with autoimmune diseases is significantly lower than that in patients with cancer, which may be related to a lower pathological B cell load [4–6,98]. Effective treatments for CRS include glucocorticoids or tolizumab, an IL-6 neutralizing antibody [97]. Close monitoring and active assessment of vital signs, neurological symptoms, and inflammatory markers during the initial post-infusion phase are essential to ensure patient safety and optimize CAR-T cell efficacy. Furthermore, the U.S. Food and Drug Administration reported that prolonged expansion and sustained activity of CAR-T cells could provoke an excessive immune response, potentially increasing the risk of developing T cell malignancies [99]. Future development of modifiable CAR structures and the integration of suicide gene safety switches will aid in achieving dynamic monitoring and the urgent elimination of T cell activity. Predictive models based on machine learning algorithms integrating multiomic inflammatory biomarkers will facilitate CRS/ICANS risk stratification management. Hypogammaglobulinemia due to B cell depletion is a long-term CAR-T therapy side effect that requires attention and may increase the risk of opportunistic infections. Risk reduction strategies include intravenous immunoglobulin replacement and prophylactic anti-infective therapy [99,100]. Notably, long-term safety data on whether CAR-T cell therapy could interfere with the underdeveloped immune systems of pediatric patients are lacking.

Nephrotoxicity associated with CAR-T cell therapy, particularly acute kidney injury (AKI), should not be overlooked in patients with AIKDs. A recent systematic review of studies of AKI after CAR-T cell therapy revealed that 22% of 694 patients with hematologic malignancies experienced AKI, with the majority recovering their renal function after symptomatic treatment [101]. Secondary fluid loss and a reduction in effective blood volume due to CRS are often considered the most common causes of concomitant AKI, as the massive release of cytokines may exacerbate damage to podocytes and endothelial cells [102,103]. The nephrotoxicity resulting from pretreatment with chemotherapeutic agents may also trigger the development of AKI [104]. However, no cases of AKI have been reported in patients with AIKDs treated with CAR-T cells, which contradicts the hypothesis that AKI is more likely to occur in patients with limited renal reserve. This discrepancy may be attributed to small sample sizes or the avoidance of conditions such as tumor lysis syndrome, which has been observed to cause AKI in patients with hematologic tumors [105]. In conclusion, pretreatment renal risk assessment as well as close post-treatment monitoring of renal function and urine output are necessary. The development of early biomarkers of kidney injury will facilitate risk prediction modeling. For patients with preexisting renal insufficiency, a more thorough evaluation of the risks and benefits of CAR-T cell therapy is necessary, and the optimal dose of the lymphocyte removal regimen should be rationally selected.

### Relapse and retreatment

Relapse after CAR-T cell therapy remains a major clinical management challenge in AIKDs. However, the relapse rate following CAR-T treatment for AIKDs remains inconsistent across studies. Evidence from hematologic malignancies suggests that the depth of initial treatment response may be a key predictor of long-term remission following CAR-T cell therapy [106–108]. In patients with SLE, relapse has been reported due to insufficient CAR-T cell infusion doses during initial treatment [37]. Optimizing CAR-T cell manufacturing conditions, increasing the number of infused cells, and employing genetic engineering strategies to develop metabolically enhanced CAR-T cells may help improve the depth of the initial response. Currently, retreatment strategies after relapse are still under exploration. Depending on the severity of relapse, short-term corticosteroids or immunosuppressive therapies may be effective in mild cases, whereas moderate to severe relapses may require consideration of a second CAR-T cell infusion. If the initial therapy was effective, reinfusion of cryopreserved CAR-T cells can be attempted, although the potential development of anti-CAR antibodies due to immunogenicity must be considered. Switching therapeutic targets (e.g., using BCMA or CD38 instead of CD19) or exploring non-CAR-T cell-based immunotherapies (such as CAR-natural killer cells) may also help reinduce durable remission. Long-term follow-up and dynamic monitoring of B cell reconstitution, autoantibody profiles, and changes in the immune microenvironment are essential for identifying patients at high risk of relapse. Furthermore, optimizing CAR constructs, such as through the use of humanized designs or the incorporation of constitutive activation domains, may help reduce the risk of relapse.

### Impact of the kidney immune microenvironment

Certain AIKDs, such as LN and IgAN, are more prone to developing a localized renal immune microenvironment, making it challenging to ensure the effective infiltration and persistence of CAR-T cells within the kidney [12,109]. However, unlike the highly immunosuppressive microenvironment of solid tumors, the immune milieu in AIKDs is characterized by active inflammation and a lack of suppressive barriers, which may facilitate tissue infiltration by CAR-T cells. Future studies are warranted to investigate whether renal CAR-T cell persistence and expansion are impaired in patients with treatment failure or disease relapse compared to those who achieve long-term remission. In the field of solid tumor immunotherapy, approaches such as coexpression of chemokine receptors (e.g., CXCR5) [110] and engineered local delivery systems (e.g., penetrative hydrogels or nanoparticle carriers) [111,112] have been explored to enhance T cell localization and tissue penetration. These strategies hold similar promise in AIKDs, especially in patients where suboptimal CAR-T efficacy is linked to the renal immune microenvironment. Additionally, CAR-engineered cell-derived exosomes represent a novel method for targeted renal delivery [113]. The development of intelligent, kidney-targeted CAR-T delivery systems may not only improve therapeutic efficacy but also reduce systemic toxicity, offering a more durable and precise immunotherapeutic approach for AIKDs.

## Conclusion and Perspectives

CAR-T cell therapy represents a breakthrough advancement over conventional B cell depletion therapies in AIKDs, enabling deeper and more sustained elimination of pathogenic B cells to induce immune reset and disease remission. However, clinical evidence remains limited regarding its long-term safety, durable efficacy, and relapse control. Challenges persist in personalized CAR-T design, optimal target selection, and precise patient stratification. Moving forward, deeper mechanistic insights into CAR-T biology, innovative production strategies, and improved risk assessment models may transform CAR-T therapy from a last-resort option to a frontline intervention for AIKDs. This evolution may ultimately establish a new treatment paradigm centered on precision immune remodeling.

## References

[1] Segelmark M, Hellmark T. Autoimmune kidney diseases. Autoimmun Rev. 2010;9(5):A366–A371. 19906361 10.1016/j.autrev.2009.11.007

[2] Foresto-Neto O, Menezes-Silva L, Leite JA, Andrade-Silva M, Câmara NOS. Immunology of kidney disease. Annu Rev Immunol. 2024;42(1):207–233. 38211945 10.1146/annurev-immunol-090122-045843

[3] Schrezenmeier E, Jayne D, Dörner T. Targeting B cells and plasma cells in glomerular diseases: Translational perspectives. J Am Soc Nephrol. 2018;29(3):741–758. 29326157 10.1681/ASN.2017040367 PMC5827591

[4] Mougiakakos D, Krönke G, Völkl S, Kretschmann S, Aigner M, Kharboutli S, Böltz S, Manger B, Mackensen A, Schett G. CD19-targeted CAR T cells in refractory systemic lupus erythematosus. N Engl J Med. 2021;385(6):567–569. 34347960 10.1056/NEJMc2107725

[5] Mackensen A, Müller F, Mougiakakos D, Böltz S, Wilhelm A, Aigner M, Völkl S, Simon D, Kleyer A, Munoz L, et al. Anti-CD19 CAR T cell therapy for refractory systemic lupus erythematosus. Nat Med. 2022;28(10):2124–2132. 36109639 10.1038/s41591-022-02017-5

[6] Müller F, Taubmann J, Bucci L, Wilhelm A, Bergmann C, Völkl S, Aigner M, Rothe T, Minopoulou I, Tur C, et al. CD19 CAR T-cell therapy in autoimmune disease—A case series with follow-up. N Engl J Med. 2024;390(8):687–700. 38381673 10.1056/NEJMoa2308917

[7] Tian D-S, Qin C, Dong M-H, Heming M, Zhou L-Q, Wang W, Cai S-B, You Y-F, Shang K, Xiao J, et al. B cell lineage reconstitution underlies CAR-T cell therapeutic efficacy in patients with refractory myasthenia gravis. EMBO Mol Med. 2024;16(4):966–987. 38409527 10.1038/s44321-024-00043-z PMC11018773

[8] Zhang C, Ma P, Qin A, Wang L, Dai K, Liu Y, Zhao J, Lu Z. Current immunotherapy strategies for rheumatoid arthritis: The immunoengineering and delivery systems. Research. 2023;6:0220. 39902178 10.34133/research.0220 PMC11789687

[9] Fischbach F, Richter J, Pfeffer LK, Fehse B, Berger SC, Reinhardt S, Kuhle J, Badbaran A, Rathje K, Gagelmann N, et al. CD19-targeted chimeric antigen receptor T cell therapy in two patients with multiple sclerosis. Med. 2024;5(6):550–558.e2. 38554710 10.1016/j.medj.2024.03.002

[10] Steinmetz OM, Velden J, Kneissler U, Marx M, Klein A, Helmchen U, Stahl RAK, Panzer U. Analysis and classification of B-cell infiltrates in lupus and ANCA-associated nephritis. Kidney Int. 2008;74(4):448–457. 18528326 10.1038/ki.2008.191

[11] Arazi A, Rao DA, Berthier CC, Davidson A, Liu Y, Hoover PJ, Chicoine A, Eisenhaure TM, Jonsson AH, Li S, et al. The immune cell landscape in kidneys of patients with lupus nephritis. Nat Immunol. 2019;20(7):902–914. 31209404 10.1038/s41590-019-0398-x PMC6726437

[12] Wang M, Rajkumar S, Lai Y, Liu X, He J, Ishikawa T, Nallapothula D, Singh RR. Tertiary lymphoid structures as local perpetuators of organ-specific immune injury: Implication for lupus nephritis. Front Immunol. 2023;14:1204777. 38022566 10.3389/fimmu.2023.1204777 PMC10644380

[13] Lou H, Ling GS, Cao X. Autoantibodies in systemic lupus erythematosus: From immunopathology to therapeutic target. J Autoimmun. 2022;132: 102861. 35872103 10.1016/j.jaut.2022.102861

[14] McAdoo SP, Pusey CD. Anti-glomerular basement membrane disease. Clin J Am Soc Nephrol. 2017;12(7):1162–1172. 28515156 10.2215/CJN.01380217 PMC5498345

[15] Hoxha E, Reinhard L, Stahl RAK. Membranous nephropathy: New pathogenic mechanisms and their clinical implications. Nat Rev Nephrol. 2022;18(7):466–478. 35484394 10.1038/s41581-022-00564-1

[16] Cui Z, Zhao MH. Anti-nephrin autoantibodies: A paradigm shift in podocytopathies. Nat Rev Nephrol. 2024;20(10):639–640. 39080046 10.1038/s41581-024-00873-7

[17] Suárez-Fueyo A, Bradley SJ, Klatzmann D, Tsokos GC. T cells and autoimmune kidney disease. Nat Rev Nephrol. 2017;13(6):329–343. 28287110 10.1038/nrneph.2017.34

[18] Colucci M, Oniszczuk J, Vivarelli M, Audard V. B-cell dysregulation in idiopathic nephrotic syndrome: What we know and what we need to discover. Front Immunol. 2022;13: Article 823204. 35140723 10.3389/fimmu.2022.823204 PMC8819007

[19] Yoshikawa T, Oguchi A, Toriu N, Sato Y, Kobayashi T, Ogawa O, Haga H, Sakurai S, Yamamoto T, Murakawa Y, et al. Tertiary lymphoid tissues are microenvironments with intensive interactions between immune cells and proinflammatory parenchymal cells in aged kidneys. J Am Soc Nephrol. 2023;34(10):1687–1708. 37548710 10.1681/ASN.0000000000000202 PMC10561819

[20] Sato Y, Silina K, van den Broek M, Hirahara K, Yanagita M. The roles of tertiary lymphoid structures in chronic diseases. Nat Rev Nephrol. 2023;19(8):525–537. 37046081 10.1038/s41581-023-00706-z PMC10092939

[21] Ambegaonkar AA, Holla P, Dizon BL, Sohn H, Pierce SK. Atypical B cells in chronic infectious diseases and systemic autoimmunity: Puzzles with many missing pieces. Curr Opin Immunol. 2022;77: Article 102227. 35724448 10.1016/j.coi.2022.102227 PMC9612402

[22] Holla P, Dizon B, Ambegaonkar AA, Rogel N, Goldschmidt E, Boddapati AK, Sohn H, Sturdevant D, Austin JW, Kardava L, et al. Shared transcriptional profiles of atypical B cells suggest common drivers of expansion and function in malaria, HIV, and autoimmunity. Sci Adv. 2021;7(22):eabg8384. 34039612 10.1126/sciadv.abg8384 PMC8153733

[23] Jacobi AM, Mei H, Hoyer BF, Mumtaz IM, Thiele K, Radbruch A, Burmester GR, Hiepe F, Dörner T. HLA-DR^high^ /CD27^high^ plasmablasts indicate active disease in patients with systemic lupus erythematosus. Ann Rheum Dis. 2010;69(1):305–308. 19196727 10.1136/ard.2008.096495

[24] Al-Aubodah T-A, Aoudjit L, Pascale G, Perinpanayagam MA, Langlais D, Bitzan M, Samuel SM, Piccirillo CA, Takano T. The extrafollicular B cell response is a hallmark of childhood idiopathic nephrotic syndrome. Nat Commun. 2023;14(1):7682. 37996443 10.1038/s41467-023-43504-8 PMC10667257

[25] Weisel FJ, Zuccarino-Catania GV, Chikina M, Shlomchik MJ. A temporal switch in the germinal center determines differential output of memory B and plasma cells. Immunity. 2016;44(1):116–130. 26795247 10.1016/j.immuni.2015.12.004 PMC4724390

[26] Hoyer BF, Moser K, Hauser AE, Peddinghaus A, Voigt C, Eilat D, Radbruch A, Hiepe F, Manz RA. Short-lived plasmablasts and long-lived plasma cells contribute to chronic humoral autoimmunity in NZB/W mice. J Exp Med. 2004;199(11):1577–1584. 15173206 10.1084/jem.20040168 PMC2211779

[27] Espeli M, Bökers S, Giannico G, Dickinson HA, Bardsley V, Fogo AB, Smith KGC. Local renal autoantibody production in lupus nephritis. J Am Soc Nephrol. 2011;22(2):296–305. 21088295 10.1681/ASN.2010050515 PMC3029902

[28] Starke C, Frey S, Wellmann U, Urbonaviciute V, Herrmann M, Amann K, Schett G, Winkler T, Voll RE. High frequency of autoantibody-secreting cells and long-lived plasma cells within inflamed kidneys of NZB/W F1 lupus mice. Eur J Immunol. 2011;41(7):2107–2112. 21484784 10.1002/eji.201041315

[29] Lacotte S, Dumortier H, Décossas M, Briand JP, Muller S. Identification of new pathogenic players in lupus: Autoantibody-secreting cells are present in nephritic kidneys of (NZBxNZW)F1 mice. J Immunol. 2010;184(7):3937–3945. 20181885 10.4049/jimmunol.0902595

[30] Cheng Q, Mumtaz IM, Khodadadi L, Radbruch A, Hoyer BF, Hiepe F. Autoantibodies from long-lived ‘memory’ plasma cells of NZB/W mice drive immune complex nephritis. Ann Rheum Dis. 2013;72(12):2011–2017. 24114925 10.1136/annrheumdis-2013-203455

[31] Kamburova EG, Koenen HJPM, Borgman KJE, ten Berge IJ, Joosten I, Hilbrands LB. A single dose of rituximab does not deplete B cells in secondary lymphoid organs but alters phenotype and function. Am J Transplant. 2013;13(6):1503–1511. 23570303 10.1111/ajt.12220

[32] Fichtner ML, Hoehn KB, Ford EE, Mane-Damas M, Oh S, Waters P, Payne AS, Smith ML, Watson CT, Losen M, et al. Reemergence of pathogenic, autoantibody-producing B cell clones in myasthenia gravis following B cell depletion therapy. Acta Neuropathol Commun. 2022;10(1):154. 36307868 10.1186/s40478-022-01454-0 PMC9617453

[33] Iwasaki T, Yoshifuji H, Kitagori K, Sumitomo S, Akizuki S, Nakashima R, Tsuji H, Hiwa R, Shirakashi M, Murakami K, et al. Memory B cells and their transcriptomic profiles associated with belimumab resistance in systemic lupus erythematosus in the maintenance phase. Front Immunol. 2025;16:1506298. 39975549 10.3389/fimmu.2025.1506298 PMC11835923

[34] Tur C, Eckstein M, Velden J, Rauber S, Bergmann C, Auth J, Bucci L, Corte G, Hagen M, Wirsching A, et al. CD19-CAR T-cell therapy induces deep tissue depletion of B cells. Ann Rheum Dis. 2025;84(1):106–114. 39874224 10.1136/ard-2024-226142

[35] Pracht K, Meinzinger J, Daum P, Schulz SR, Reimer D, Hauke M, Roth E, Mielenz D, Berek C, Côrte-Real J, et al. A new staining protocol for detection of murine antibody-secreting plasma cell subsets by flow cytometry. Eur J Immunol. 2017;47(8):1389–1392. 28608550 10.1002/eji.201747019

[36] Tedder TF. CD19: A promising B cell target for rheumatoid arthritis. Nat Rev Rheumatol. 2009;5(10):572–577. 19798033 10.1038/nrrheum.2009.184

[37] Wang W, He S, Zhang W, Zhang H, DeStefano VM, Wada M, Pinz K, Deener G, Shah D, Hagag N, et al. BCMA-CD19 compound CAR T cells for systemic lupus erythematosus: A phase 1 open-label clinical trial. Ann Rheum Dis. 2024;83(10):1304–1314. 38777376 10.1136/ard-2024-225785

[38] Qin C, Zhang M, Mou DP, Zhou LQ, Dong MH, Huang L, Wang W, Cai SB, You YF, Shang K, et al. Single-cell analysis of anti-BCMA CAR T cell therapy in patients with central nervous system autoimmunity. Sci Immunol. 2024;9(95):eadj9730. 38728414 10.1126/sciimmunol.adj9730

[39] Stoiber S, Cadilha BL, Benmebarek MR, Lesch S, Endres S, Kobold S. Limitations in the design of chimeric antigen receptors for cancer therapy. Cells. 2019;8(5):472. 31108883 10.3390/cells8050472 PMC6562702

[40] Chung JB, Brudno JN, Borie D, Kochenderfer JN. Chimeric antigen receptor T cell therapy for autoimmune disease. Nat Rev Immunol. 2024;24(11):830–845. 38831163 10.1038/s41577-024-01035-3 PMC12176013

[41] Tschumi BO, Dumauthioz N, Marti B, Zhang L, Lanitis E, Irving M, Schneider P, Mach JP, Coukos G, Romero P, et al. CART cells are prone to Fas- and DR5-mediated cell death. J Immunother Cancer. 2018;6(1):71. 30005714 10.1186/s40425-018-0385-z PMC6045821

[42] Cao X, Chen J, Li B, Dang J, Zhang W, Zhong X, Wang C, Raoof M, Sun Z, Yu J, et al. Promoting antibody-dependent cellular phagocytosis for effective macrophage-based cancer immunotherapy. Sci Adv. 2022;8(11):eabl9171. 35302839 10.1126/sciadv.abl9171 PMC8932662

[43] Khawar MB, Afzal A, Si Y, Sun H. Steering the course of CAR T cell therapy with lipid nanoparticles. J Nanobiotechnology. 2024;22(1):380. 38943167 10.1186/s12951-024-02630-1 PMC11212433

[44] Zheng Z, Li S, Liu M, Chen C, Zhang L, Zhou D. Fine-tuning through generations: Advances in structure and production of CAR-T therapy. Cancers. 2023;15(13):3476. 37444586 10.3390/cancers15133476 PMC10340266

[45] Rurik JG, Tombácz I, Yadegari A, Méndez Fernández PO, Shewale SV, Li L, Kimura T, Soliman OY, Papp TE, Tam YK, et al. CAR T cells produced in vivo to treat cardiac injury. Science. 2022;375(6576):91–96. 34990237 10.1126/science.abm0594 PMC9983611

[46] Li YR. Managing allorejection in off-the-shelf CAR-engineered cell therapies. Mol Ther. 2024.10.1016/j.ymthe.2024.11.03539600090

[47] Degagné É, Donohoue PD, Roy S, Scherer J, Fowler TW, Davis RT, Reyes GA, Kwong G, Stanaway M, Vicena VL, et al. High-specificity CRISPR-mediated genome engineering in anti-BCMA allogeneic CAR T cells suppresses allograft rejection in preclinical models. Cancer Immunol Res. 2024;12(4):462–477. 38345397 10.1158/2326-6066.CIR-23-0679 PMC10985478

[48] Wang X, Wu X, Tan B, Zhu L, Zhang Y, Lin L, Xiao Y, Sun A, Wan X, Liu S, et al. Allogeneic CD19-targeted CAR-T therapy in patients with severe myositis and systemic sclerosis. Cell. 2024;187(18):4890–4904.e9. 39013470 10.1016/j.cell.2024.06.027

[49] Chen Z, Shu J, Hu Y, Mei H. Synergistic integration of mRNA-LNP with CAR-engineered immune cells: Pioneering progress in immunotherapy. Mol Ther. 2024;32(11):3772–3792. 39295145 10.1016/j.ymthe.2024.09.019 PMC11573621

[50] Wu J, Wu W, Zhou B, Li B. Chimeric antigen receptor therapy meets mRNA technology. Trends Biotechnol. 2024;42(2):228–240. 37741706 10.1016/j.tibtech.2023.08.005

[51] Rossi M, Breman E. Engineering strategies to safely drive CAR T-cells into the future. Front Immunol. 2024;15:1411393. 38962002 10.3389/fimmu.2024.1411393 PMC11219585

[52] Dimitri A, Herbst F, Fraietta JA. Engineering the next-generation of CAR T-cells with CRISPR-Cas9 gene editing. Mol Cancer. 2022;21(1):78. 35303871 10.1186/s12943-022-01559-z PMC8932053

[53] Karadimitris A. Cord blood CAR-NK cells: Favorable initial efficacy and toxicity but durability of clinical responses not yet clear. Cancer Cell. 2020;37(4):426–427. 32289266 10.1016/j.ccell.2020.03.018

[54] Arias J, Yu J, Varshney M, Inzunza J, Nalvarte I. Hematopoietic stem cell- and induced pluripotent stem cell-derived CAR-NK cells as reliable cell-based therapy solutions. Stem Cells Transl Med. 2021;10(7):987–995. 33634954 10.1002/sctm.20-0459 PMC8235144

[55] Zhou Z, Chen Y, Ba Y, Xu H, Zuo A, Liu S, Zhang Y, Weng S, Ren Y, Luo P, et al. Revolutionising cancer immunotherapy: Advancements and prospects in non-viral CAR-NK cell engineering. Cell Prolif. 2025;58(4): Article e13791. 39731215 10.1111/cpr.13791 PMC11969250

[56] Chen Y, Xin Q, Zhu M, Qiu J, Luo Y, Li R, Wei W, Tu J. Exploring CAR-macrophages in non-tumor diseases: Therapeutic potential beyond cancer. J Adv Res. 2025.10.1016/j.jare.2025.01.00439756574

[57] Capsomidis A, Benthall G, Van Acker HH, Fisher J, Kramer AM, Abeln Z, Majani Y, Gileadi T, Wallace R, Gustafsson K, et al. Chimeric antigen receptor-engineered human gamma delta T cells: Enhanced cytotoxicity with retention of cross presentation. Mol Ther. 2018;26(2):354–365. 29310916 10.1016/j.ymthe.2017.12.001 PMC5835118

[58] Zhang Q, Lu W, Liang CL, Chen Y, Liu H, Qiu F, Dai Z. Chimeric antigen receptor (CAR) Treg: A promising approach to inducing immunological tolerance. Front Immunol. 2018;9:2359. 30369931 10.3389/fimmu.2018.02359 PMC6194362

[59] Reincke SM, von Wardenburg N, Homeyer MA, Kornau H-C, Spagni G, Li LY, Kreye J, Sánchez-Sendín E, Blumenau S, Stappert D, et al. Chimeric autoantibody receptor T cells deplete NMDA receptor-specific B cells. Cell. 2023;186(23):5084–5097.e18. 37918394 10.1016/j.cell.2023.10.001

[60] Oh S, Mao X, Manfredo-Vieira S, Lee J, Patel D, Choi EJ, Alvarado A, Cottman-Thomas E, Maseda D, Tsao PY, et al. Precision targeting of autoantigen-specific B cells in muscle-specific tyrosine kinase myasthenia gravis with chimeric autoantibody receptor T cells. Nat Biotechnol. 2023;41(9):1229–1238. 36658341 10.1038/s41587-022-01637-z PMC10354218

[61] Ellebrecht CT, Bhoj VG, Nace A, Choi EJ, Mao X, Cho MJ, di Zenzo G, Lanzavecchia A, Seykora JT, Cotsarelis G, et al. Reengineering chimeric antigen receptor T cells for targeted therapy of autoimmune disease. Science. 2016;353(6295):179–184. 27365313 10.1126/science.aaf6756 PMC5343513

[62] Seifert L, Riecken K, Zahner G, Hambach J, Hagenstein J, Dubberke G, Huber TB, Koch-Nolte F, Fehse B, Tomas NM. An antigen-specific chimeric autoantibody receptor (CAAR) NK cell strategy for the elimination of anti-PLA2R1 and anti-THSD7A antibody-secreting cells. Kidney Int. 2024;105(4):886–889. 38309682 10.1016/j.kint.2024.01.021

[63] Solé C, Royo M, Sandoval S, Moliné T, Gabaldón A, Cortés-Hernández J. Precise targeting of autoantigen-specific B cells in lupus nephritis with chimeric autoantibody receptor T cells. Int J Mol Sci. 2024;25(8):4226. 38673811 10.3390/ijms25084226 PMC11050013

[64] Guo Y, Xie YQ, Gao M, Zhao Y, Franco F, Wenes M, Siddiqui I, Bevilacqua A, Wang H, Yang H, et al. Metabolic reprogramming of terminally exhausted CD8^+^ T cells by IL-10 enhances anti-tumor immunity. Nat Immunol. 2021;22(6):746–756. 34031618 10.1038/s41590-021-00940-2 PMC7610876

[65] Zhao Y, Chen J, Andreatta M, Feng B, Xie YQ, Wenes M, Wang Y, Gao M, Hu X, Romero P, et al. IL-10-expressing CAR T cells resist dysfunction and mediate durable clearance of solid tumors and metastases. Nat Biotechnol. 2024;42(11):1693–1704. 38168996 10.1038/s41587-023-02060-8

[66] Heuser-Loy C. Interleukin-10 gives exhausted chimeric antigen receptor (CAR) T cells a second breath. Cancer Commun. 2024;44(7):787–790. 10.1002/cac2.12575 PMC11260757 38863177

[67] Ye L, Park JJ, Peng L, Yang Q, Chow RD, Dong MB, Lam SZ, Guo J, Tang E, Zhang Y, et al. A genome-scale gain-of-function CRISPR screen in CD8 T cells identifies proline metabolism as a means to enhance CAR-T therapy. Cell Metab. 2022;34(4):595–614.e14. 35276062 10.1016/j.cmet.2022.02.009 PMC8986623

[68] Zhang Q, Hresko ME, Picton LK, Su L, Hollander MJ, Nunez-Cruz S, Zhang Z, Assenmacher CA, Sockolosky JT, Garcia KC, et al. A human orthogonal IL-2 and IL-2Rβ system enhances CAR T cell expansion and antitumor activity in a murine model of leukemia. Sci Transl Med. 2021;13(625):eabg6986. 34936380 10.1126/scitranslmed.abg6986 PMC9116279

[69] Stavrou M, Philip B, Traynor-White C, Davis CG, Onuoha S, Cordoba S, Thomas S, Pule M. A rapamycin-activated caspase 9-based suicide gene. Mol Ther. 2018;26(5):1266–1276. 29661681 10.1016/j.ymthe.2018.03.001 PMC5993966

[70] Gargett T, Brown MP. The inducible caspase-9 suicide gene system as a “safety switch” to limit on-target, off-tumor toxicities of chimeric antigen receptor T cells. Front Pharmacol. 2014;5:235. 25389405 10.3389/fphar.2014.00235 PMC4211380

[71] Kansal R, Richardson N, Neeli I, Khawaja S, Chamberlain D, Ghani M, Ghani QUA, Balazs L, Beranova-Giorgianni S, Giorgianni F, et al. Sustained B cell depletion by CD19-targeted CAR T cells is a highly effective treatment for murine lupus. Sci Transl Med. 2019;11(482):eaav1648. 30842314 10.1126/scitranslmed.aav1648 PMC8201923

[72] Jin X, Xu Q, Pu C, Zhu K, Lu C, Jiang Y, Xiao L, Han Y, Lu L. Therapeutic efficacy of anti-CD19 CAR-T cells in a mouse model of systemic lupus erythematosus. Cell Mol Immunol. 2021;18(8):1896–1903. 32472023 10.1038/s41423-020-0472-1 PMC8322088

[73] Arbuckle MR, McClain MT, Rubertone MV, Scofield RH, Dennis GJ, James JA, Harley JB. Development of autoantibodies before the clinical onset of systemic lupus erythematosus. N Engl J Med. 2003;349(16):1526–1533. 14561795 10.1056/NEJMoa021933

[74] Charras A, Smith E, Hedrich CM. Systemic lupus erythematosus in children and young people. Curr Rheumatol Rep. 2021;23(3):20. 33569643 10.1007/s11926-021-00985-0 PMC7875946

[75] Krickau T, Naumann-Bartsch N, Aigner M, Kharboutli S, Kretschmann S, Spoerl S, Vasova I, Völkl S, Woelfle J, Mackensen A, et al. CAR T-cell therapy rescues adolescent with rapidly progressive lupus nephritis from haemodialysis. Lancet. 2024;403(10437):1627–1630. 38642568 10.1016/S0140-6736(24)00424-0

[76] He X, Hu B, Zhang Y, Liu F, Li Q, Zheng C, Shen J, Yang Z, Wang J, Ma D, et al. Treatment of two pediatric patients with refractory systemic lupus erythematosus using CD19-targeted CAR T-cells. Autoimmun Rev. 2025;24(1): Article 103692. 10.1016/j.autrev.2024.103692 39561867

[77] Lodka D, Zschummel M, Bunse M, Rousselle A, Sonnemann J, Kettritz R, Höpken UE, Schreiber A. CD19-targeting CAR T cells protect from ANCA-induced acute kidney injury. Ann Rheum Dis. 2024;83(4):499–507. 38182404 10.1136/ard-2023-224875 PMC10958264

[78] Taddeo A, Khodadadi L, Voigt C, Mumtaz IM, Cheng Q, Moser K, Alexander T, Manz RA, Radbruch A, Hiepe F, et al. Long-lived plasma cells are early and constantly generated in New Zealand black/New Zealand white F1 mice and their therapeutic depletion requires a combined targeting of autoreactive plasma cells and their precursors. Arthritis Res Ther. 2015;17(1):39. 25889236 10.1186/s13075-015-0551-3 PMC4411657

[79] Müller F, Wirsching A, Hagen M, Völkl S, Tur C, Raimondo MG, Taubmann J, Bucci L, Zhang L, Kretschmann S, et al. BCMA-CAR T-cells in a patient with relapsing idiopathic inflammatory myositis after CD19-CAR T-cells. Nat Med. 2025. 10.1038/s41591-025-03718-3 PMC12176613 40245922

[80] Markmann C, Bhoj VG. On the road to eliminating long-lived plasma cells-“are we there yet?”. Immunol Rev. 2021;303(1):154–167. 34351644 10.1111/imr.13015

[81] Fontenot JD, Gavin MA, Rudensky AY. Foxp3 programs the development and function of CD4^+^CD25^+^ regulatory T cells. Nat Immunol. 2003;4(4):330–336. 12612578 10.1038/ni904

[82] Kasagi S, Zhang P, Che L, Abbatiello B, Maruyama T, Nakatsukasa H, Zanvit P, Jin W, Konkel JE, Chen WJ. In vivo–generated antigen-specific regulatory T cells treat autoimmunity without compromising antibacterial immune response. Sci Transl Med. 2014;6(241):241ra78. 10.1126/scitranslmed.3008895 24944193

[83] Beheshti SA, Shamsasenjan K, Ahmadi M, Abbasi B. CAR Treg: A new approach in the treatment of autoimmune diseases. Int Immunopharmacol. 2022;102: Article 108409. 34863655 10.1016/j.intimp.2021.108409

[84] Ma D-H, Yang X-D, Hua Q-J, Hou Y-L, Liu Y, Xu Q-Y, Lian L, Zhou Y-L, Guo M-H. Changes and significance of Treg and Th17 in adult patients with primary membranous nephropathy. Clin Nephrol. 2021;96(3):155–164. 33993908 10.5414/CN110333

[85] Free ME, Bunch DO, McGregor JA, Jones BE, Berg EA, Hogan SL, Hu Y, Preston GA, Jennette JC, Falk RJ, et al. Patients with antineutrophil cytoplasmic antibody-associated vasculitis have defective Treg cell function exacerbated by the presence of a suppression-resistant effector cell population. Arthritis Rheum. 2013;65(7):1922–1933. 23553415 10.1002/art.37959 PMC3717615

[86] Jin LW, Ye HY, Xu XY, Zheng Y, Chen Y. MiR-133a/133b inhibits Treg differentiation in IgA nephropathy through targeting FOXP3. Biomed Pharmacother. 2018;101:195–200. 29494956 10.1016/j.biopha.2018.02.022

[87] Zhong H, Liu Y, Xu Z, Liang P, Yang H, Zhang X, Zhao J, Chen J, Fu S, Tang Y, et al. TGF-β-induced CD8^+^CD103^+^ regulatory T cells show potent therapeutic effect on chronic graft-versus-host disease lupus by suppressing B cells. Front Immunol. 2018;9:35. 29441062 10.3389/fimmu.2018.00035 PMC5797539

[88] Kluger MA, Melderis S, Nosko A, Goerke B, Luig M, Meyer MC, Turner JE, Meyer-Schwesinger C, Wegscheid C, Tiegs G, et al. Treg17 cells are programmed by Stat3 to suppress Th17 responses in systemic lupus. Kidney Int. 2016;89(1):158–166. 26466322 10.1038/ki.2015.296

[89] Ooi JD, Snelgrove SL, Engel DR, Hochheiser K, Ludwig-Portugall I, Nozaki Y, O’Sullivan KM, Hickey MJ, Holdsworth SR, Kurts C, et al. Endogenous foxp3^+^ T-regulatory cells suppress anti-glomerular basement membrane nephritis. Kidney Int. 2011;79(9):977–986. 21248715 10.1038/ki.2010.541

[90] Shen BL, Qu QS, Miao SZ, Liu BL, Liu RY, Gu DF. Study on the effects of regulatory T cells on renal function of IgAN rat model. Eur Rev Med Pharmacol Sci. 2015;19(2):284–288. 25683943

[91] Ahmad SB, Appel GB. Antigens, antibodies, and membranous nephropathy: A decade of progress. Kidney Int. 2020;97(1):29–31. 31901352 10.1016/j.kint.2019.10.009

[92] Wang T, Marken J, Chen J, Tran VB, Li QZ, Li M, Cerosaletti K, Elkon KB, Zeng X, Giltiay NV. High *TLR7* expression drives the expansion of CD19^+^CD24^hi^CD38^hi^ transitional B cells and autoantibody production in SLE patients. Front Immunol. 2019;10:1243. 31231380 10.3389/fimmu.2019.01243 PMC6559307

[93] Sang A, Danhorn T, Peterson JN, Rankin AL, O’Connor BP, Leach SM, Torres RM, Pelanda R. Innate and adaptive signals enhance differentiation and expansion of dual-antibody autoreactive B cells in lupus. Nat Commun. 2018;9(1):3973. 30266981 10.1038/s41467-018-06293-z PMC6162205

[94] Neelapu SS, Dickinson M, Munoz J, Ulrickson ML, Thieblemont C, Oluwole OO, Herrera AF, Ujjani CS, Lin Y, Riedell PA, et al. Axicabtagene ciloleucel as first-line therapy in high-risk large B-cell lymphoma: The phase 2 ZUMA-12 trial. Nat Med. 2022;28(4):735–742. 35314842 10.1038/s41591-022-01731-4 PMC9018426

[95] Neelapu SS, Dickinson M, Munoz J, Ulrickson ML, Thieblemont C, Oluwole OO, Herrera AF, Ujjani CS, Lin Y, Riedell PA, et al. Primary analysis of ZUMA-12: A phase 2 study of axicabtagene ciloleucel (axi-cel) as first-line therapy in patients with high-risk large B-cell lymphoma (LBCL). Blood. 2021;138(Supplement 1):739.

[96] Brown ART, Gutierrez C. Comments regarding “ASBMT consensus grading for cytokine release syndrome and neurologic toxicity associated with immune effector cells”. Biol Blood Marrow Transplant. 2019;25(6):e209–e210. 30862467 10.1016/j.bbmt.2019.02.027

[97] Morris EC, Neelapu SS, Giavridis T, Sadelain M. Cytokine release syndrome and associated neurotoxicity in cancer immunotherapy. Nat Rev Immunol. 2022;22(2):85–96. 34002066 10.1038/s41577-021-00547-6 PMC8127450

[98] Bergmann C, Müller F, Distler JHW, Györfi AH, Völkl S, Aigner M, Kretschmann S, Reimann H, Harrer T, Bayerl N, et al. Treatment of a patient with severe systemic sclerosis (SSc) using CD19-targeted CAR T cells. Ann Rheum Dis. 2023;82(8):1117–1120. 37147112 10.1136/ard-2023-223952 PMC10359520

[99] Cappell KM, Kochenderfer JN. Long-term outcomes following CAR T cell therapy: What we know so far. Nat Rev Clin Oncol. 2023;20(6):359–371. 37055515 10.1038/s41571-023-00754-1 PMC10100620

[100] Sharma N, Reagan PM, Liesveld JL. Cytopenia after CAR-T cell therapy—A brief review of a complex problem. Cancers. 2022;14(6):1501. 35326654 10.3390/cancers14061501 PMC8946106

[101] Yang Y, Luo K, Xu G. Acute kidney injury following chimeric antigen receptor T-cell therapy: Epidemiology, mechanism and prognosis. Clin Immunol. 2024;266: Article 110311. 38996858 10.1016/j.clim.2024.110311

[102] Gupta S, Seethapathy H, Strohbehn IA, Frigault MJ, O’Donnell EK, Jacobson CA, Motwani SS, Parikh SM, Curhan GC, Reynolds KL, et al. Acute kidney injury and electrolyte abnormalities after chimeric antigen receptor T-cell (CAR-T) therapy for diffuse large B-cell lymphoma. Am J Kidney Dis. 2020;76(1):63–71. 31973908 10.1053/j.ajkd.2019.10.011 PMC7311244

[103] León-Román J, Iacoboni G, Bermejo S, Carpio C, Bolufer M, García-Carro C, Sánchez-Salinas M, Alonso-Martínez C, Bestard O, Barba P, et al. Transient acute kidney injury after chimeric antigen receptor T-cell therapy in patients with hematological malignancies. Clin Kidney J. 2024;17(3):sfae027. 38500492 10.1093/ckj/sfae027 PMC10946657

[104] Schett G, Müller F, Taubmann J, Mackensen A, Wang W, Furie RA, Gold R, Haghikia A, Merker PA, Caricchio R, et al. Advancements and challenges in CAR T cell therapy in autoimmune diseases. Nat Rev Rheumatol. 2024;20(9):531–544. 39107407 10.1038/s41584-024-01139-z

[105] Biró E, Erdélyi D, Varga P, Sinkó M, Bartyik K, Kovács G, Ottóffy G, Vincze F, Szegedi I, Kiss C, et al. Daily serum phosphate increase as early and reliable indicator of kidney injury in children with leukemia and lymphoma developing tumor lysis syndrome. Pediatr Nephrol. 2023;38(9):3117–3127. 36943467 10.1007/s00467-023-05923-z PMC10432329

[106] Cappell KM, Sherry RM, Yang JC, Goff SL, Vanasse DA, McIntyre L, Rosenberg SA, Kochenderfer JN. Long-term follow-up of anti-CD19 chimeric antigen receptor T-cell therapy. J Clin Oncol. 2020;38(32):3805–3815. 33021872 10.1200/JCO.20.01467 PMC7655016

[107] Schuster SJ, Tam CS, Borchmann P, Worel N, McGuirk JP, Holte H, Waller EK, Jaglowski S, Bishop MR, Damon LE, et al. Long-term clinical outcomes of tisagenlecleucel in patients with relapsed or refractory aggressive B-cell lymphomas (JULIET): A multicentre, open-label, single-arm, phase 2 study. Lancet Oncol. 2021;22(10):1403–1415. 34516954 10.1016/S1470-2045(21)00375-2

[108] Frey NV, Gill S, Hexner EO, Schuster S, Nasta S, Loren A, Svoboda J, Stadtmauer E, Landsburg DJ, Mato A, et al. Long-term outcomes from a randomized dose optimization study of chimeric antigen receptor modified T cells in relapsed chronic lymphocytic leukemia. J Clin Oncol. 2020;38(25):2862–2871. 32298202 10.1200/JCO.19.03237 PMC8265376

[109] Luo R, Chang D, Zhang N, Cheng Y, Ge S, Xu G. T follicular helper cells in tertiary lymphoid structure contribute to renal fibrosis by IL-21. Int J Mol Sci. 2023;24(16):12535. 37628716 10.3390/ijms241612535 PMC10454845

[110] Hui X, Farooq MA, Chen Y, Ajmal I, Ren Y, Xue M, Ji Y, du B, Wu S, Jiang W. A novel strategy of co-expressing CXCR5 and IL-7 enhances CAR-T cell effectiveness in osteosarcoma. Front Immunol. 2024;15:1462076. 39450160 10.3389/fimmu.2024.1462076 PMC11499113

[111] Suraiya AB, Evtimov VJ, Truong VX, Boyd RL, Forsythe JS, Boyd NR. Micro-hydrogel injectables that deliver effective CAR-T immunotherapy against 3D solid tumor spheroids. Transl Oncol. 2022;24: Article 101477. 35905640 10.1016/j.tranon.2022.101477 PMC9334344

[112] Zhang F, Stephan SB, Ene CI, Smith TT, Holland EC, Stephan MT. Nanoparticles that reshape the tumor milieu create a therapeutic window for effective T-cell therapy in solid malignancies. Cancer Res. 2018;78(13):3718–3730. 29760047 10.1158/0008-5472.CAN-18-0306 PMC6030470

[113] Fan M, Liu H, Yan H, Che R, Jin Y, Yang X, Zhou X, Yang H, Ge K, Liang XJ, et al. A CAR T-inspiring platform based on antibody-engineered exosomes from antigen-feeding dendritic cells for precise solid tumor therapy. Biomaterials. 2022;282: Article 121424. 35196606 10.1016/j.biomaterials.2022.121424

